# Avoiding Accidents and Failures in Anesthesia

**Published:** 1906-01-15

**Authors:** Egbert T. Loeffler


### AVOIDING ACCIDENTS AND FAILURES IN ANESTHESIA.

BY EGBERT T. LOEFFLER, B.S., D.D.S.

So long as we are human, we must expect occasionally to meet with failure or accident in any undertaking or practice, and in spite of every precaution. In many cases, however, yes we might add in the majority of cases, failures and accidents may be traced to some error of observation, of judgment or of technic. And yet in many instances a most searching examination would fail to reveal the error by avoiding which the failure or accident might have been avoided or prevented. Some of these cases might be designated as unavoidable. It would, however, be difficult to imagine a combination of circumstances, which by careful study and forethought could not have been prevented.

Some of the unpleasant features have come to every man who has had any considerable amount of experience in the administration of anesthetics, and yet there are certain preventive measures, which if, observed, will certainly lessen their frequency.

The lesson of every failure seems obvious. It ought to teach us to search into the history of the patient and determine by careful examination of every part of the body, if possible, and find out whether or not some predisposition does not exist to explain the disastrous result. It seems almost as if the anesthetic had a tendency to bring out the unsuspected latent condition.

The measures and means used for preventing undesirable calamities may have been carefully noted and yet accidents will happen. Dr. M. H. Richardson in one of his articles says: "Since sudden and unforseen death in people not known to be ill, and those who are attending to their daily labors is common enough, it would appear that the surgeon should be familiar with the way in which such a death occurs, inasmuch as it would be almost certain to occur in the same manner after operative work. By paying rigid attention to the personal history of the patient and to all the resources of diagnosis, by the recognition of the conditions which may predispose and the symptoms which portend, the means which may prevent, and the course which may retrieve disaster, we should be able to exclude some of these cases as inoperable.

Lochte has recently written on this subject, and his paper is based on autopsies of one hundred and one cases of sudden death. "It is not fair," he states, "to claim, as is popularly done, that such cases are stricken down in the bloom of health. In a majority it would have been possible for an examination to have determined that something was wrong." This writer evidently seems to indicate that some patients are aware that their physical condition is not up to par, but make light of it. Apparently they were in good condition.

Dr. J. L. Dickey, in his interesting paper on angina pectoris, a few years ago, gives out the following facts:

The first cause, in frequency, of such deaths as referred to is disease of the heart, of the coronary arteries in thirty-one victims. Of great importance is the fact that these people were young, only one being over fifty years of age; not one had reached the fatal age of fifty-five.

The second cause, in frequency, is alcoholism with twenty-five cases, the great majority being chronic.

The third class the author styles "walking cases" of acute infectious diseases, with twelve victims.

The rest of the material included seven cerebral apoplexis, seven cases of aneurisms and some unclassified. In all the males predominated. From the above mentioned statistical report, we can readily see that failure and disaster are apt to occur in the practice of any man, and often when least expected.

Of course we all understand that one of the gravest conditions of human life is that of anesthesia. The boundary line between life and death is so closely approached and yet so rarely crossed that it seems absolutely astonishing. This makes the fact very plain, that only to the most skilled in the use of those agents which abolish sensibility should be entrusted their administration.

But you ask how can some of these disastrous results be avoided or prevented? The answer to this all-important question has already been hinted at. An answer in detail to every phase of this question would consume too much time and would certainly not come within the meaning and purpose of this discussion.

First of all then, I would say that all those who intend to make a practice of giving anesthetics, either as a specialty or in their own private practice, should thoroughly familiarize themselves with the normal functions of the human body, and more particularly with those vital functions respiration and the circulation, of which the heart is the prime factor.

In order to understand disease of organs in perverted conditions we must, of course, have a definite conception or clear picture of the healthy or the physiological. A perfect knowledge of this kind will enable us to detect the slightest deviation from the normal and it at once leads us to look for the cause.

A thorough physical diagnosis is important. The pulse, its peculiarities and its significance with regard to the circulation. The various conditions of the pupil and what they mean. The nerves which control the iris and especially an intimate knowledge of the vital nerve centres in the Medulla.

Lastly, but not least by any means, a clear understanding as to how these centres are effected by operations on the fifth pair of nerves.

In studying the action of anesthetics or of any drugs, for that matter, most of us know very well that we base their action on theory first, then on physiological action, as derived from experimentation on lower animals, and finally the crowning test is always the clinical application, for we do not always find that laboratory experiments tally with clinical experience, even after we have eliminated all physchic factors.

There still remain personal equations, idiosyncracies, and natural differences between artificial diseases of animals and those of the human kind, all producing a difference in results. As an illustration, I might say that it has not yet been proven just how general anesthesia is produced or what part the blood plays in its production, and whether or not the hæmoglobin can be considered as a factor. In regard to this point it seems reasonable to suppose that a general anesthetic would be more injurious to a patient whose blood is low in these constituents than to an individual with a normal amount of hæmoglobin. But we do not know this action, so we must, for the present at least, rely on our clinical experience in drawing our conclusions.

For those of us, therefore, who have just cause for apprehension in this regard and who have escaped anesthetic dangers, it would be a wise plan to obtain more knowledge on the subject and by all means more practical experience.

It certainly should be urged upon those who wish to become proficient in the administration of so potent an agent as a general anesthetic that, unless they fully realize the responsibility and danger assumed, they can never hope to attain even a foundation of what has been so aptly termed, "The art of Anesthesia."

Competent authorities hold that the reckless as well as the timid are equally dangerous, the first because he does not seem to fully realize that his meager knowledge and training alone stand between patient and failure or disaster, or possibly death; the second because he seems to have no faith in his accomplishment, though he may have the very best preparation to assure his fitness.

Practically before beginning every operation, the apparatus to be used should be carefully examined to see that it is in proper working order. The anesthetic should be tested, inhaled through the inhaler to see that it has the desired effect. An alternative anesthetic should be at hand should the exigencies of the case require it. Mouth props, tongue forceps, sponges and holders, a mouth opener, preparation of strychnine, aromatic spirits of ammonia and a good working hypodermic syringe. Superarenal extracts, the powder in three grains capsules, are excellent in case the patient is able to swallow.

For an all-around restorative, oxygen which can be had so conveniently in cylinders, is one of the most useful agents to have in the equipment. It need hardly be mentioned that all instruments, inhaling apparatus, etc., should be thoroughly and efficiently cleaned both before and after using.

In conclusion I might say, especially to the timid, that frequent re-reading of articles on the physiological action and symptoms of anesthetics as given in reliable texts is an absolute necessity and would have a wholesome tendency to relieve, if not prevent many otherwise embarrassing failures and accidents.

